# Evaluation of Stable LifeAct-mRuby2- and LAMP1-NeonGreen Expressing A549 Cell Lines for Investigation of *Aspergillus fumigatus* Interaction with Pulmonary Cells

**DOI:** 10.3390/ijms22115965

**Published:** 2021-05-31

**Authors:** Natalia Schiefermeier-Mach, Violetta Moresco, Stephan Geley, Lea Heinrich, Lukas Lechner, Heidi Oberhauser, Susanne Perkhofer

**Affiliations:** 1 FH Gesundheit/Health University of Applied Sciences Tyrol, Innrain 98, 6020 Innsbruck, Austria; violettamoresco@gmail.com (V.M.); lea.heinrich@fhg-tirol.ac.at (L.H.); lukas.lechner@fhg-tirol.ac.at (L.L.); heidi.oberhauser@fhg-tirol.ac.at (H.O.); susanne.perkhofer@fhg-tirol.ac.at (S.P.); 2 Institute of Pathophysiology, Medical University of Innsbruck, Innrain 52, 6020 Innsbruck, Austria; stephan.geley@i-med.ac.at

**Keywords:** LifeAct, LAMP1, stable cell lines, microscopy, live-cell imaging, *Aspergillus fumigatus*, conidia internalization, cell-pathogen interactions

## Abstract

Inhaled *Aspergillus fumigatus* spores can be internalized by alveolar type II cells. Cell lines stably expressing fluorescently labeled components of endocytic pathway enable investigations of intracellular organization during conidia internalization and measurement of the process kinetics. The goal of this report was to evaluate the methodological appliance of cell lines for studying fungal conidia internalization. We have generated A549 cell lines stably expressing fluorescently labeled actin (LifeAct-mRuby2) and late endosomal protein (LAMP1-NeonGreen) following an evaluation of cell-pathogen interactions in live and fixed cells. Our data show that the LAMP1-NeonGreen cell line can be used to visualize conidia co-localization with LAMP1 in live and fixed cells. However, caution is necessary when using LifeAct-mRuby2-cell lines as it may affect the conidia internalization dynamics.

## 1. Introduction

The lung is constantly exposed to external environmental factors including a range of microbes. Immunocompromised patients are at high risk for acquiring pulmonary infectious diseases, frequently caused by *Aspergillus fumigatus*, which can lead to aspergilloma and eventually life-threatening invasive aspergillosis. In addition, hypersensitivity reactions can cause allergic bronchopulmonary aspergillosis, a serious and hard-to-treat disease. Thus, understanding the specific mechanisms of the fungal infection remains essential. After inhalation, *A. fumigatus* spores (conidia) can reach terminal airspaces and contact alveolar epithelial cells. Previous studies showed that *A. fumigatus* conidia are internalized by both professional phagocytes and even alveolar type II cells, which are normally not phagocytic [1,2].

The infectious process begins with the adhesion of *A. fumigatus* conidia to the host cell plasma membrane that initiates formation of pseudopods, which engulf and finally endocytose conidia [2]. During internalization, an actin ring encircles the conidia-containing endosomes, which further mature into late endosomes/phagolysosomes [1,3,4], a mechanism similar to the receptor-mediated endo- or phagocytosis [5]. Lung macrophages efficiently internalize bound conidia and various cultured airway epithelial cells may internalize conidia to some extent [1,6,7,8]. One of the receptors that might be responsible for conidia uptake was recently identified, a C-type lectin receptor (MelLec) that recognizes melanin in the cell wall of conidia [9]. MelLec is expressed in endothelial cells as well as myeloid cells in the lungs and mutation of this receptor in mice and humans causes loss of an important innate immune barrier against this pathogen [9]. Thus, specific recognition and cellular uptake is an important antifungal mechanism. Upon endocytosis, conidia are degraded in phagolysosomes but many details of this process need to be resolved and it is unclear whether and how conidia can escape degradation.

Conidia phagocytosis is an actin-dependent process, regulation of actin polymerization and depolymerization by actin-binding proteins is essential [10,11,12,13]. Many pathogenic microbes manipulate the actin cytoskeleton in order to increase their cellular uptake, facilitate their replication, and promote their dispersion. Early studies proposed a transient F-actin localization to the phagosomal membrane that disappears after pathogen internalization is completed, thus enabling fusion of phagosomes with endosomes and lysosomes [7]. More recent studies showed that actin assembly is required for phagosome-endosome fusion [8,13]. Swollen conidia or application of a major mycotoxin of *A. fumigatus*, gliotoxin, induced phosphorylation of actin-binding protein cofilin [14,15]. This toxin also promoted actin cytoskeleton rearrangement and conidia internalization [15].

Late endosomal protein LAMP1 is commonly used as a marker of late endosomes and phagolysosomes [8]. Conidia internalization into LAMP1-positive vesicles was shown in macrophages, several epithelial cell lines, and to a lower extent in primary lung cells [1,4,8]. Studies in macrophages found that the number of LAMP1-positive phagosomes increased during the course of *A. fumigatus* infection until all ingested conidia had a LAMP1-positive ring around them [8]. Conidia internalization and trafficking in non-phagocytic cells differs from that in macrophages: Less than 60% of conidia was found in a late endosomal compartment by 24 h in A549 cells and a significant amount of internalized conidia remained viable [3].

Spatial organization of intracellular components during conidia internalization can be visualized using fluorescently labeled proteins and live cell microscopy allowing the measurement of internalization and degradation kinetics. Moreover, cell lines stably expressing fluorescently labeled components of endocytic pathway represent valuable tools for studying cell-pathogen interactions. In this study, we generated and evaluated conidia internalization in A549 stable cell lines expressing fluorescently labeled actin (LifeAct-mRuby2) and late endosomal protein (LAMP1-NeonGreen).

## 2. Results and Discussion

### 2.1. Generation of A549 Cells Lines Stably Expressing LifeAct-mRuby2 and LAMP1

**Figure 1:**
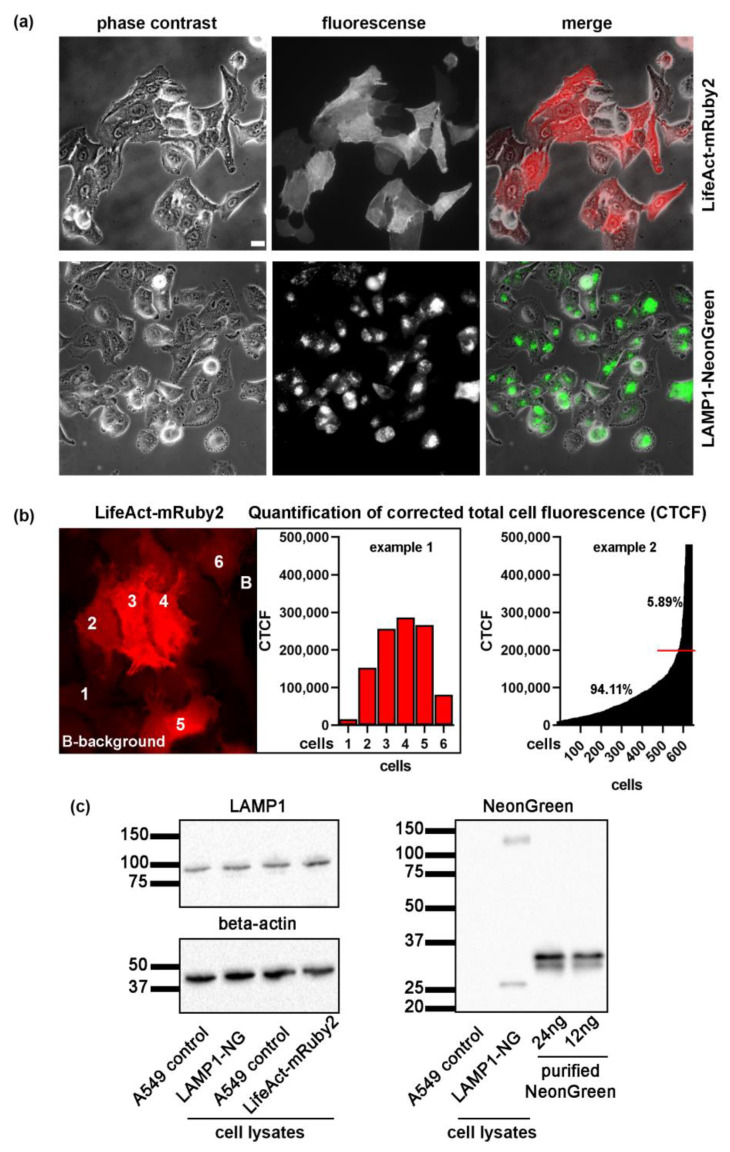
(**a**) Generation of A549 cell lines stably expressing LifeAct-mRuby2 (in red) and LAMP1-NG (NeonGreen in green). Scale bar 20 µm. (**b**) Quantification of corrected total cell fluorescence of LifeAct-Ruby2 cells. Example 1 illustrates the image and quantification of six individual cells, value for each cell is depicted. Example 2 shows measurements of 628 cells from a single experiment. The red line depicts a cut-off point for high CTCF values reflecting strongly expressing Ruby2-positive cells. (**c**) Expression of LAMP1, beta-actin, and NeonGreen in generated cell lines.

In order to investigate the *A. fumigatus* conidia interaction with lung cells, we have generated cell lines stably expressing LifeAct and LAMP1. LifeAct is the most common fluorescent tool for actin visualization in living cells that was used in more than 1800 studies [16,17]. It represents a short 17-amino-acid peptide from Saccharomyces cerevisiae actin-binding protein 140 (Abp140p) fused to a fluorescent protein [18]. LifeAct binds to G-actin with higher affinity compared to filamentous actin, it has a small size (30 kDa), and does not strongly interfere with actin dynamics [16,19]. Therefore, LifeAct is a valuable tool to study actin-dependent cellular processes such as conidia internalization. Here, we generated a novel color variant, LifeAct-mRuby2, which is a fusion of the yeast Abp140 [19] peptide to mRuby2 red fluorescent protein mRuby2 [20]. LAMP1 fused to fluorescent NeonGreen [21] was previously shown to be a proper marker for late endosomes/lysosomes [22]. Using these markers, generated stable cell lines can therefore be used to visualize both early and late stages of conidia phagocytosis. Both genes were stably integrated into the host cells genomes by lentiviral transduction. Expressed fluorescent proteins exhibited low to middle levels of protein expression and proper localization to cytoplasmic actin (LifeAct) and LAMP1 (LAMP1-NeonGreen) according to previous studies (Figure 1a). In order to exclude the effect of LifeAct overexpression, quantification of corrected total fluorescence was performed as previously described [23,24,25]. Only 5.68% of LifeAct expressing cells showed a strong fluorescent signal (Figure 1b). No actin rings could be revealed in these cells and they were excluded from all further analysis. For the LAMP1-NeonGreen, we have performed Western blotting using the NeonGreen purified protein and anti-NeonGreen antibodies. Around 120,000 cells were loaded per gel line and band intensity was compared to a given amount of purified NeonGreen (Figure 1c). We did not reveal a strong overexpression of NeonGreen, LAMP1, and beta-actin in any of the cell lysates.

### 2.2. A. fumigatus Conidia Internalization in A549 Cells Stably Expressing LifeAct-mRuby2

**Figure 2:**
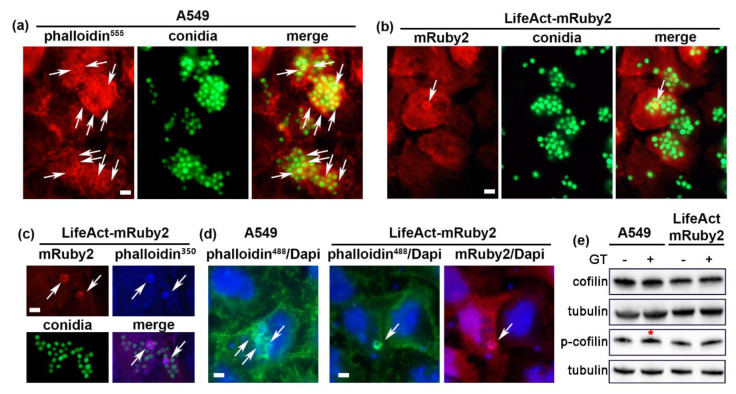
Infection of A549 and LifeAct-mRuby2 cells with *A. fumigatus* results in formation of actin rings around internalized fungal conidia. (**a**) Actin rings in A549 cells (white arrows). Immunofluorescence: Phalloidin-Alexa 555 (actin) in red, GFP-conidia in green, (**b**) actin rings in LifeAct-mRuby2-expressing cells (white arrows). GFP-conidia in green, (**c**) actin rings in LifeAct-mRuby2-expressing cells (white arrows). Immunofluorescence: Phalloidin-Alexa 350 (actin) in blue, GFP-conidia in green, (**d**) actin rings (white arrows) in A549 and LifeAct-mRuby2 cells visualized by phalloidin-Alexa 488 (green), Dapi in blue. (**e**) Expression and phosphorylation of cofilin in cells treated with gliotoxin. Red asterisk points on phosphorylation of cofilin after treatment with gliotoxin in control A549 cells. GT: Gliotoxin. Scale bar 10 µm. See also Video S1 in Supplementary Material.

Next, we investigated the early stages of conidia internalization in the newly generated cell lines and compared it to the original A549 cells (further called “A549” cells). Swollen GFP-expressing *A. fumigatus* conidia were applied on cells for 2 h to allow attachment. Unattached conidia were washed with PBS following 1 h of internalization prior to either cell imaging or cell fixation and immunofluorescence staining (see Material and Methods). In agreement with the previous studies [1,3,4], we have observed the formation of actin rings surrounding *A. fumigatus* conidia in fixed control A549 cells as visualized by phalloidin. However, when 10^5^ or 10^6^ cfu/mL conidia was added to LifeActRed-expressing cells (further called “LifeActRed cells”) and live-cell imaging was performed, we did not observe the formation of actin rings in these cells (data not shown). Only when an artificially high amount of conidia was applied (10^7^ cfu/mL), rings were also visible in LifeAct-expressing cells as visualized by mRuby2-fluorescence (Figure 2a,b and Video S1 in Supplementary Material). Surprisingly, the percentage of conidia surrounded by actin ring in cells stably expressing LifeAct-mRuby2 cells was significantly lower than in control A549 cells (8.54 ± 3.20% vs. 23.89 ± 5.28%, value < 0.005) (Figure 2a,c). To ensure that LifeAct-mRuby2 visualized all the actin rings formed, we additionally stained conidia-infected LifeAct-mRuby2 cells with phalloidin AlexaFluo350 (Figure 2c) and AlexaFluo488 (Figure 2d). Phalloidin staining revealed the same actin structures as visualized by mRuby2 fluorescence. These experiments were repeated with *A. fumigatus* isolated from a patient where conidia were stained with the Calcofluor White reagent or Dapi and no difference to GFP-expressing conidia was observed (data not shown).

Recent publications suggest that LifeAct may not be a universal marker for actin imaging. LifeAct-CFP and LifeAct-mRFP constructs did not visualize cofilin-bound actin rods in the cell nuclei during cell stress [17,26,27]. Further studies also suggested that introducing fluorescently labeled LifeAct in living cells may cause changes in F-actin distribution [28] and actin-dependent biological processes such as endocytosis, cytokinesis, and cell migration in a concentration-dependent manner [17,27]. The expression EGFP-LifeAct resulted in a decreased velocity of the actin patches assembly/disassembly in yeast cells and impaired the ability of contractile rings to assemble and constrict [17]. An actin-binding protein, cofilin, was proposed to be affected by the LifeAct expression. Cofilin plays essential roles in cytoskeleton dynamics and internalization of pathogenic microbes since it catalyzes both the polymerization and depolymerization of actin [12]. Flores et al. showed that the transient transfection of hMSC cells with the LifeAct-TagGFP2 construct increased the expression and phosphorylation of cofilin [27]. It was hypothesized that LifeAct, when bound to F-actin, may induce conformational changes in the actin filaments structure and consequently make it incompatible with cofilin binding, resulting in inhibition of the cofilin-induced actin depolymerization thus reducing actin dynamics [17,27]. On the other hand, Munisie et al. proposed that cofilin binding to F-actin prevents binding of the LifeAct protein [26]. In this case, one would expect to see actin rings formed around internalized conidia that are negative for LifeAct-mRuby2. We did not observe phalloidin-stained actin rings that would not express LifeAct-mRuby2 (Figure 2c,d). Therefore, our results favor the hypothesis that LifeAct rather than cofilin causes F-actin conformational changes preventing the binding of the latter. Interestingly, previous studies showed that the swollen conidia of *A. fumigatus* induced alterations in cofilin phosphorylation on serine 3 in A549 cells. This phosphorylation is known to inhibit the severing activity of cofilin and decrease actin dynamics. Both down- and upregulation of cofilin phosphorylation inhibited *A. fumigatus* internalization [14]. The application of gliotoxin, induced cofilin phosphorylation in A549 cells as well as promoted actin cytoskeleton rearrangement and conidia internalization [15]. Thus, fine-tuning of cofilin activity by phosphorylation caused by gliotoxin might be an important parameter influencing *A. fumigatus* internalization [15]. We have treated A549 and LifeAct-mRuby2 cells with 50 ng/mL gliotoxin for 30 min [15] and visualized cofilin/phospho-cofilin by immunoblotting. A slight decrease in total cofilin expression in LifeAct-mRuby2 cells was observed which was not affected by the gliotoxin application in our experimental settings (Figure 2e). Moreover, gliotoxin caused a minor increase in cofilin phosphorylation in control A549 cells (red asterisk in Figure 2e) which was not detected in LifeAct-mRuby2 cells. Our data suggest that the expression and/or fine-turning of cofilin activation could partly explain the decreased formation of actin rings in LifeAct-mRuby2 cells. However, we did not detect major changes in cofilin expression/phosphorylation suggesting alternative mechanism(s).

Taken together, our data suggest that the viral transduction of LifeAct-mRuby2 used in our study may not be optimal for *A. fumigatus* conidia internalization studies, as it inhibits the formation of actin rings around conidia. Certainly, we cannot directly compare our results to the transient transfections used in other studies. The transient nature of LifeAct (over)expression may have a different effect on cofilin expression and the switch between active unphosphorylated and inactive phosphorylated form. Cofilin expression might have “adapted” during cell culture and passaging to allow cell viability, since we also did not find many cells overexpressing LifeAct (Figure 1b).

### 2.3. A. fumigatus Conidia Internalization in A549 Cells Stably Expressing LAMP1

**Figure 3:**
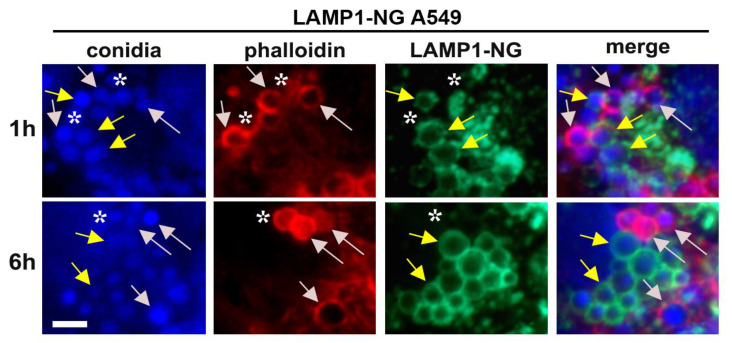
*A. fumigatus* conidia internalization by A549 cell lines stably expressing LAMP1-NG. Conidia internalization for the indicated periods. Immunofluorescence: Dapi (conidia in blue), phalloidin (actin in red), LAMP1-NG in green. Grey arrows point to conidia co-localized with actin rings. Yellow arrows point to conidia co-localized with LAMP1-NG-rings. Asterisks identify the conidia not surrounded by actin or LAMP1, h: hours. Scale bar 10 µm. See also Video S2 in Supplementary Material.

Further, we tested the LAMP1-NeonGreen A549 cell line (“LAMP1-NG cells”) to investigate conidia internalization into the late endosomal compartment. We performed a live video microscopy and experiments in fixed cells using a similar experimental setup as in Figure 2, with an extended time course of internalization. The infection of cells with 10^5^ cfu/mL of *A. fumigatus* conidia caused co-localization of LAMP1 with conidia and further formation of GFP-positive vesicles around conidia (Figure 3, Video S2 in Supplementary Material). When we applied a high conidia concentration (10^7^ cfu/mL) and performed phalloidin staining in fixed LAMP-1-NG cells, the percentage of actin rings was similar to the control A549 cells (26.49 ± 5.01% vs. 23.89 ± 5.28%). Our results are in agreement with previous studies showing similar dynamics of conidia trafficking in A549 cells using LAMP1 antibodies [1,3]. Therefore, the viral transduction of LAMP1-NG in A549 cells did not affect conidia internalization into late endosomes.

To summarize, phagocytosis of *A. fumigatus* can be visualized in A549 cells using lentiviral transduction of fluorescently labeled LifeAct and LAMP1 proteins and the impact of fluorescent proteins on the dynamics of conidia internalization has to be carefully assessed to avoid artefacts. Our data confirm and extend previous studies showing that introducing LifeAct may affect the kinetics of cellular processes: Caution is necessary when using LifeAct-mRuby2 cell lines for imaging of cell-pathogen interactions.

## 3. Materials and Methods

### 3.1. Reagents, Cell Culture, and Generation of Cell Lines

The RPMI-1640 medium (RPMI-XA), fetal bovine serum (FBS), L-glutamine, penicillin/streptomycin, and phosphate buffered saline (PBS) were from Capricorn Scientific (Ebsdorfergrund, Germany). Lentiviral particles were produced using the HEK-293 cell line, which was transfected with two packaging plasmids psPAX2, VSV-G, and LifeAct-mRuby2 or LAMP1-NeonGreen [22] plasmids. In addition, 48 h later the medium containing viral particles was harvested, sterile filtered (pore size: 0.2 µm), and used to infect the A549 cells. Viral particles were left for 24 h, the successful transfection and localization of viral constructs to actin cytoskeleton (for LifeAct) or late endosomes (for LAMP-1) was observed by the fluorescent microscope. Puromycin dihydrochlorid (2 ug/mL, Lactan, Carl Roth, Karlsruhe, Germany) was added to the growth medium. LifeAct-mRuby2 was generated by fusing the coding sequence of the actin binding domain (aa 1–17) of yeast Abp140 [19] to the 5′ end of the red fluorescent protein mRuby2 [20] by PCR and subsequent subcloning into retroviral pQCXIN (Clontech Laboratories, Inc., Mountain View, CA, USA) using the Gateway technology. LAMP1-NeonGreen was described in [22]. Anti-NeonGreen antibodies were raised in rabbits against hexahistidine tagged NeonGreen expressed in and purified from *E. coli* BL21 [DE3] (Davids Biotechnologie, Regensburg, Germany).

### 3.2. Preparation and Application of A. fumigatus Conidia

The GFP-expressing *A. fumigatus* strain FGSC A1258 gGFP and *A. fumigatus* clinical isolate were grown and harvested as described before [29]. Freshly harvested conidia (10^5^–10^7^ cfu/mL) were kept in RPMI-1640 in a shaking incubator at 37 °C, 160 rpm for 2 h to obtain the swollen conidia. The swollen conidia were applied on cells for 2 h to allow attachment. The unattached conidia were extensively washed out with PBS and cells were further incubated for 1, 3, and 6 h following fixation.

### 3.3. Immunofluorescence

Cells were grown on 12-mm-diameter glass coverslips until 80–100% confluence and immunofluorescence was described previously [30]. In short, cells were fixed in 4% PFA (#104005, Merck, Darmstadt, Germany) and permeabilized with 0.5% Saponin (Sigma-Aldrich, Vienna, Austria) for 10 min followed by washing in PBS. Blocking was performed in PBS containing 10% BSA (Sigma-Aldrich, Vienna, Austria), 0.5% Saponin, and 5% goat serum (Vector Laboratories, Burlingame, CA, USA). Phalloidin was diluted 1:5000 in a blocking solution and incubated for 1 h followed by washing in PBS and mounting in Moviol (Sigma-Aldrich, Vienna, Austria). The BD Calcofluor White reagent (Thermo Fischer Scientific, Vienna, Austria) and Dapi (1:20,000, Sigma-Aldrich, Vienna, Austria) were used according to the manufacturer’s protocol, while Phalloidin AlexaFluor555 (#A34055), Phalloidin AlexaFluor488 (#A12379), and Phalloidin AlexaFlour350 (#A22281, Thermo Fischer Scientific, Vienna, Austria) were used for actin staining. Microscopy images were taken using an Oxion Inverso Microscope, precooled CCD camera (Euromex Microscopen, BD Arnhem, The Netherlands) and the ImageFocus 4, v.2.8 software (Euromex Microscopen, BD Arnhem, The Netherlands). Z-stack images and live videos were taken using inverse Zeiss Axiovert 200 M (Carl Zeiss, Jena, Germany) equipped with a CCD camera (CoolSNAP HQ2; Photometrics, Tucson, AZ, USA) and the AxioVision release 4.5 SP1 software (Carl Zeiss, Jena, Germany). Live video microscopy was performed using the Zeiss setup at 37 °C and 5% CO2 incubation conditions. Figures and movies were prepared in Fiji Software [31], Adobe Photoshop CC (version 19.1.7, Adobe Systems Incorporated, San Jose, CA, USA), and GraphPad Prism v8.0 (GraphPad Software, San Diego, CA, USA). Quantification of corrected total fluorescence was performed using the Fiji Software (based on ImageJ 1.51n, maintained by Laboratory for Optical and Computational Instrumentation at the University of Wisconsin-Madison, Madison, WI, USA, [31]) according to the previously described method [23,24,25].

### 3.4. Conidia Quantification

For each performed experiment, 2.2–2.6 × 10^6^ cells were treated with 3 × 10^6^ or 3 × 10^7^ conidia (1.15–1.36 or 11.54–13.64 conidia per cell accordingly). For conidia quantification, each imaging plane was photographed three times with a Z-plane shift. Z-planes were overlaid using the Z-Project and maximum projection functions in Fiji Software (based on ImageJ 1.51n, maintained by Laboratory for Optical and Computational Instrumentation at the University of Wisconsin-Madison, Madison, WI, USA, [31]). Quantifications of 10 sections pro-experiment were independently performed by two co-authors (V.M. and L.H.), each experiment was repeated at least 3 times. The ANOVA test in Microsoft Excel (Microsoft Corp., Redmond, WA, USA) was used for statistical analysis. A *p*-value < 0.005 was considered as significant.

### 3.5. Western Blotting

Immunoblotting was performed using the standard procedure [32]. The gel chamber, nitrocellulose membrane, and standard reagents were from Bio-Rad Laboratories (Vienna, Austria). Total cell lysates were prepared using RIPA (Bio-Rad Laboratories, Vienna, Austria) on ice, followed by 10 s sonication (QSONICA Q55 Sonicator, Thermo Fischer Scientific, Vienna, Austria) and 20 min centrifugation at 14,000 rpm. Lysates mixed with a loading buffer (#1610747, Bio-Rad Laboratories, Wien, Austria) were separated on Mini-PROTEAN TGXTM gels (#4561034, Bio-Rad Laboratories, Vienna, Austria). Lysates from approximately 120,000 cells were loaded per lane. The semi-dry transfer was performed using PierceG2Fast Blotter (Thermo Fischer Scientific, Vienna, Austria), the Xpress Blotting buffer was from SERVA Electrophoresis. Blocking was performed in 5% BSA/TPBS/0.5% Tween (1 h incubation at room temperature). The primary antibodies (overnight incubation at 4 °C or 1 h incubation at room temperature for tubulin and beta-actin antibodies) were as follows: LAMP1 (#55273-1-AP, Cell Signaling Technology Europe, Leiden, The Netherlands), beta-actin (#8457T, Proteintech Europe, Manchester, UK anti-cofilin (D3F9)-XP (#5175, 1:1000, Cell Signaling Technology Europe, Leiden, The Netherlands), anti-phospho-cofilinSer3-77G2 (#3313, 1:1000, Cell Signaling Technology Europe, Leiden, The Netherlands), and anti-tubulin (#T5168-100 UL, 1:5000, Sigma-Aldrich, Vienna, Austria). Secondary HRP-conjugated antibodies (1 h incubation at room temperature) were as follows: Goat anti-rabbit (#G21234) and goat anti-mouse (#A10551) (both 1:10,000, Thermo Fischer Scientific, Vienna, Austria). Membranes were developed/visualized using the Clarity MaxTM Western ECL (#1705060), ChemiDocTmXRS+ Imager and Image LabTM software (all from Bio-Rad Laboratories, Vienna, Austria). Gliotoxin (MedChemExpress, Monmouth Junction, NJ, USA) was applied for 30 min, with a concentration of 50 ng/mL as previously described [15]. The NeonGreen purified protein was produced in the laboratory of Stephan Geley as follows: NeonGreen expression was induced from plasmid pNCS-NeonGreen in 1 L of *E. coli* BL21 [DE3] culture at OD600 = 0.6 using 0.1 mM IPTG (Lactan, Carl Roth, Karlsruhe, Germany) for 3 h at 37 °C. After pelleting, cells were resuspended in 50 mL PBS and lysed in the presence of 1 mM PMSF (Lactan, Carl Roth, Karlsruhe, Germany) and 0.5 mg/mL lysozyme (Lactan, Carl Roth, Karlsruhe, Germany). After centrifugation, the supernatant was applied to Ni-NTA agarose beads for 2 h at 4 °C. Beads were washed in a 10 mL IMAC 5 buffer (300 mM NaCl, 50 mM Tris-HCl pH 7.5, 1 mM PMSF, 5 mM benzamidine (Lactan, Carl Roth, Karlsruhe, Germany), and 5 mM imidazole (Sigma-Aldrich, Vienna, Austria), all standard reagents were from Sigma-Aldrich, Vienna, Austria) followed by a wash in IMAC 10 (300 mM NaCl, 50 mM Tris-HCl pH 7.5, 1 mM PMSF, 5 mM benzamidine, 10 mM imidazole). The recombinant NeonGreen protein was eluted in IMAC 200 (300 mM NaCl, 50 mM Tris-HCl pH 7.5, 200 mM imidazole), and positive fractions were pooled for dialysis 2 times against 2 L of PBS. The protein concentration was measured using the Bradford assay (Bio-Rad Laboratories, Vienna, Austria).

## Data Availability

All data needed to support the conclusions in the paper are present in the paper and supplementary material. All data and materials used in the analysis are available upon request to the corresponding author.

## Supplementary Materials

Supplementary materials can be found at https://www.mdpi.com/article/10.3390/ijms22115965/s1. Video S1 (for Figure 2): Shows formation of actin ring around GFP-A. fumigatus conidia in LifeAct-mRuby2 cells. Imaging in RFP- and GFP-channels. Time is indicated in hours:minutes. Video S2 (for Figure 3): Shows co-localization of LAMP1-NG with A. fumigatus conidia and formation of LAMP1-positive vesicles around internalized conidia. Imaging in phase contrast (left) and GFP-channel (right). Red arrows point to examples of LAMP1-NG co-localization with conidia. Time is indicated in hours:minutes.
